# St. Louis Medical Society

**Published:** 1882-05

**Authors:** Wm. Dickinson, A. H. Ohmann-Dumesnil

**Affiliations:** President; St. Louis, Mo.; Hall of the St. Louis Medical Society; Recording Secretary; St. Louis, Mo.; Hall of the St. Louis Medical Society


### ARTICLE XVI.

ST. LOUIS, MO., April 1, 1882,
HALL OF THE ST. LOUIS MEDICAL SOCIETY.

The following resolutions were adopted by the St. Louis Medical Society, April 1, 1882:

*Resolved,* That the St. Louis Medical Society, while it desires to accord the broadest freedom to medical investigation, and recognizes fully the right of individuals to form and hold private opinions, hereby declares that it regards with disfavor any steps taken to lessen or obliterate the distinctions and safeguards between an honorable practice of medicine founded upon science and that founded upon any of the current delusions and exclusive medical systems of the day.

*Resolved,* That a copy of this resolution be forwarded by the Oorresponding Secretary to the New York State Medical Society, to the Permanent Secretary of the American Medical Association and to several specified medical journals.

WM. DICKINSON, M. D., President.
A. H. OHMANN-DUMESNIL, M. D., Recording Secretary.

